# Immunomodulatory Effects of Aerobic Training in Obesity

**DOI:** 10.1155/2011/308965

**Published:** 2011-03-10

**Authors:** Thomas Nickel, Henner Hanssen, Ingrid Emslander, Verena Drexel, Gernot Hertel, Arno Schmidt-Trucksäss, Claudia Summo, Zeljka Sisic, Marius Lambert, Eva Hoster, Martin Halle, Michael Weis

**Affiliations:** ^1^Medizinische Klinik und Poliklinik I, Campus Grosshadern, Ludwig-Maximilians-Universität München, Marchioninistr.15, 81377 Munich, Germany; ^2^Department of Prevention and Sports Medicine, Technische Universität München, Klinikum rechts der Isar (MRI), 80809 Munich, Germany; ^3^Sports Medicine, Institute of Exercise and Health Sciences, University Basel, 4003 Basel, Switzerland; ^4^Institute for Medical Informatics Biometry and Epidemiology, Ludwig-Maximilians-Universität München, 81377 Munich, Germany

## Abstract

*Introduction*. Physical inactivity and obesity are independent risk factors for atherosclerosis. We analyzed the immunomodulatory capacity of 10-week intensified exercise training (ET) in obese and lean athletes. Markers of the innate immune response were investigated in obese (ONE: ET≤40 km/week) and lean athletes (LNE: ET≤40 km/week and LE: ET≥55 km/week). *Methods*. Circulating dendritic cells (DC) were analyzed by flow-cytometry for BDCA-1/-2-expression. TLR-2/-4/-7 and MyD88 were analyzed by RT-PCR and Western blot. Circulating oxLDL levels were analyzed by ELISA. *Results*. BDCA-1 expression at baseline was lower in ONE compared to both other groups (ONE 0.15%; LNE 0.27%; LE 0.33%; *P* < .05), but significantly increased in ONE after training (+50%; *P* < .05). In contrast, BDCA-2 expression at baseline was higher in ONE (ONE 0.25%; LNE 0.11%; LE 0.09%; *P* < .05) and decreased in ONE after the 10-week training period (−27%; *P* < .05). Gene activations of TLR-4 and TLR-7 with corresponding protein increase were found for all three groups (*P* < .01/*P* < .05) compared to pre training. A reduction of oxLDL levels was seen in ONE (−61%; *P* < .05). *Conclusions*. Intensified exercise induces an increase of BDCA-1+ DCs and TLR-4/-7 in obese athletes. We hereby describe new immune modulatory effects, which—through regular aerobic exercise—modulate innate immunity and pro-inflammatory cytokines in obesity.

## 1. Introduction

Atherosclerosis is a chronic, smoldering immune-mediated disease. Environmental factors, including diet and physical activity, play a central role for the balance of a normal immune homeostasis [1]. Physical activity is associated with a reduced risk of cardiovascular disease, cardiovascular death, and total mortality (−30–45%) in men [2]. Beside these cardiovascular effects, also incidence, mortality, and relapse of cancer are reduced by regular activity [3]. 

Obesity, an ubiquitous disease, is associated with increased cardiovascular events as well as a higher incidence of neoplasms [4, 5]. Obese subjects suffer from subclinical inflammation, which has been suggested to induce for example insulin resistance and metabolic syndrome [6]. Obesity is associated with increased plasma levels of proinflammatory cytokines such as TNF-*α* and IL-6 [7]. Moreover, adiponectin levels are inversely correlated with the grade of obesity and also with the risk of colon cancer [8] but also adiponectin levels increase by exercise [9]. 

These immunomodulatory changes found in obesity could also affect the defence capacity of the immune system making obese subjects more susceptible to infection [10]. Current research efforts are being made to address the existing relationship between obesity and immunocompetence and its impact on the long-term development of cardiovascular diseases [11]. 

The beneficial effects of physical activity-induced reduced cardiovascular risk are, in part, mediated by an exercise-induced amelioration of inflammation [7, 12]. Cross-sectional and longitudinal studies on the coherence of exercise and the immune response demonstrate long-term anti-inflammatory effects [7, 12, 13]. 

Dendritic cells (DCs) are on one hand crucial mediators of the innate immune response and represent on the other hand a link to the adaptive immune system. DCs are omnipresent, highly potent antigen presenting cells, and able to prime naive T-cells [14, 15]. BDCA-1+ DCs (DC-1) trigger a Th-1- and BDCA-2+ DCs (DC-2) a Th-2-immune-response [16–18]. 

Toll-like receptors (TLRs), which are expressed on different types of immune-cells, are crucial mediators of the innate immune response [19]. The most important adapter protein for TLRs is MyD88 which activates the transcription factor NF-*κ*B [20]. 

Relatively little is known about the impact of exercise on DC-differentiation and for TLR-expression even if they take up an important pace in initiation and modulation of the immune defense [15, 21]. 

In this study, we analyzed the immunomodulatory capacity of a 10-week exercise program, focusing on TLR-expression, DC-differentiation, and systemic inflammatory markers. We tested the hypothesis, whether regular exercise training affects DC-differentiation and TLR-expression differently in obese verses lean runners with different levels of cardiovascular fitness.

## 2. Methods

### 2.1. Subjects

Sixty amateur marathon runners intending to participate in the 2007 Munich marathon were recruited by a study appeal in a local newspaper and by written invitations sent to local running clubs. The study was approved by the hospital's ethics committee of the Technical University of Munich. Recruitment was limited to healthy male marathon runners aged 30–60 years who had run at least a half-marathon in the previous 3 years and who had no cardiovascular risk factor other than obesity. The candidates volunteered for an individual tailored, supervised training program. All athletes gave written informed consent. The sample size was restricted to sixty subjects in order to ensure optimal individual supervised exercise training over a time period of 10 weeks.

### 2.2. Study Design and Exercise Training Protocol

The study was approved by the hospital's ethics committee of the Technical University of Munich. Exclusion criteria consisted of known coronary or structural heart disease, insulin-dependent diabetes mellitus, drug treatment for type 2 diabetes or hypertension, hyperlipoproteinemia, renal dysfunction, chronic inflammatory, and musculoskeletal disorder. Subjects were divided into two age-matched groups depending on the extensiveness of training: ONE and LNE were scheduled for ≤40 km/week and LE was scheduled for ≥55 km/week. Premarathon exercise training in ONE and LNE was only sporadic, whereas LE was characterized by continuous steady state training throughout the year.

Within the first group of non-elite runners, subjects were divided into obese (body mass index [BMI] ≥30 kg/m^2^ and waist circumference [WC] ≥102 cm) and nonobese participants. A medical history, a general questionnaire, and a physical examination, including determination of body fat composition (caliper measurement), standardized routine blood pressure measurements, and fasting (overnight) blood samples, were obtained 2–5 days before and after the training program. All participants fasted before the blood draws and acute bouts of exercise were avoided 2 days prior to the examination. Anthropometry was performed by a single experienced examiner.

Participants took part in a 10-week endurance developed by sports scientists according to the current guidelines [22]. The exercise program of all groups consisted of continuous aerobic exercise and interval training including concomitant warm-up and cool-down periods with a gradual increase in duration and intensity during the course of the 10-week training program. Before the start of the training program, each athlete performed a symptom-limited treadmill ergometry (6 km/h, increase by 1 km/h every 3 min) to determine the individual anaerobic threshold (IAT), allowing individual classification of training intensity and fitness levels. Accordingly, an individual training program for each participant was prescribed with regard to current fitness levels. The intensity of the training was given by individual target heart rates. Participants used heart rate monitors. Supervised exercise training by sports scientists was offered four times a week under medical supervision. Training was documented with respect to intensity, duration, and kilometres run per week by a written protocol. After the 10-week training program, the treadmill ergometry was repeated in order to quantify the improvement of the individual fitness level.

### 2.3. Flow-Cytometer Analysis of Peripheral Blood Mononuclear Cells

Donor blood was prepared as described before [23]. Antibodies were matched with iso-type-controls (Mouse-*γ*2a-(FITC)/-*γ*1(PE)-FastImmune; BD; USA). A total of 250.000 events were acquired and analyzed using Cellquest (BD, Belgium) [23]. For determining subpopulations of DCs we analyzed BDCA-1/*‒*2 expression (Miltenyibiotec, Germany) following the protocol of Narbutt et al. [24]

### 2.4. Total RNA Isolation and Real-Time PCR

TLR-2/-4/-7 and MyD88 expression were analyzed using RT-PCR. mRNA isolation and cDNA synthesis were performed according to the instructions provided by the manufacturer and based on our previous publication [23]. The quantitative RT-PCR system provides optimal performance with SYBR Green primers (Qiagen, Germany). RT-PCR was performed in the ABI PRISMTM 7700 System (Applied Biosystems, Germany). Data analysis was performed using the delta-delta ct method [23, 25]. The different primer sequences (MWG-Biotech AG, Germany) of the analyzed receptors are as follow. 

*GAPDH:* 1 5^'^-CGG AGT CAA CGG ATT TGG TCG TAT-3^'^; 2 5^'^- AGC CTT CTC CAT GGT GGT GAA GAC-3^'^; *TLR-2:* 1 5^'^- CCA CTT GCC AGG AAT GAA GT-3^'^; 2 5^'^- GAT GCC TAC TGG GTG GAG AA-5^'^; *TLR-4:* 1 5^'^- TCC ATA AAA GCC GAA AGG TG-3^'^; 2 5^'^- GAT ACC AGC ACG ACT GCT CA-3^'^; *TLR-7:* 1 5^'^-TTA CCT GGA TGG AAA CCA GCT ACT-3^'^; 2 5^'^-TCA AGG CTG AGA AGC TGT AAG CTA-3^'^; *MyD88:* 1 5^'^-GCA CAT GGG CAC ATA CAG AC-3^'^; 2 5^'^-GAC ATG GTT AGG CTC CCT CA-3^'^; *NF*κ*B:* 1 5^'^-TGG AGT CTG GGA AGG ATT TG-3^'^; 2 5^'^- CGA AGC TGG ACA AAC ACA GA-3^'^.

### 2.5. Western Blot

PBMCs were isolated and their protein extracted according to the instructions provided by the manufacturer and based on our previous publication [15].

The anti-MyD88 (mouse) (Santa Cruz, USA) was diluted 1 : 200, the anti-TLR-2 (mouse) (IMGENEX, USA) was diluted 1 : 500, the anti-TLR-4 (mouse) (IMGENEX, USA) 1 : 150, and the anti-TLR-7 (rabbit) (IMGENEX, USA) 1 : 150, respectively. All antibodies were incubated at 4°C overnight. Beta actin (goat) (1 : 500, Santa Cruz, USA) was used as the internal standard to ensure that equal amounts of protein were loaded. 

The antigen-antibody complex was visualized using anti-mouse HRP 1 : 2500 (Santa Cruz, USA) for MyD88, TLR-2 and TLR-4, anti-rabbit HRP 1 : 1000 (Santa Cruz, USA) for TLR-7, and anti-goat HRP 1 : 5000 (Santa Cruz, USA) for beta actin. An enhanced chemiluminescence detection system (ECL-Pierce, Invitrogen; USA) was developed using the X-Omat (Kodak; USA). Quantitative analysis of Western blots by densitometry was carried out using the histogram function in Photoshop 7.0 software. All values were normalised to the beta actin loading control.

### 2.6. Plasma Concentration of oxLDL, Adiponectin, IL-6, and TNF-*α* by ELISA

Plasma tubes were centrifuged and the plasma was fractionated and frozen at −80°C. Samples were defrosted and oxLDL, adiponectin, IL-6, and TNF-*α* levels were examined by using cytokine-specific ELISA kit according to the manufacturers' instructions (oxLDL: Immunoteck, Germany; adiponektin: RayBio, USA; IL-6: Bender Med-Systems, Austria; TNF-*α*: Biosource, USA). The probes were distributed in duplicates on the plates and intra-assay and interassay coefficients of variation were ≤10%.

### 2.7. Statistical Analysis

Data are presented as mean +/− standard-deviation of the mean (SDM) and by boxplots representing the interquartile range (25th to 75th percentile) around the median (dark line in each box). The Kolmogorov-Smirnov test was used to determine whether or not the data were normally distributed. Data that were not normally distributed were analyzed using the Wilcoxon signed Rank Test for paired samples. Differences between values before and after training were compared between the three groups by means of the Kruskal-Wallis test. Adopting a closed testing procedure, only if the Kruskal-Wallis test was significant, pairwise comparisons were added post-hoc using the Mann-Whitney U test. Differences between means were considered significant with *P* < .05 and highly significant with *P* < .01. SPSS (Version 16, IBM-USA) was used for statistical analysis.

## 3. Results

### 3.1. Study Group

 From the originally recruited 20 probands per group, 15 in the obese-non-elite (ONE) group and 16 in the lean-non-elite (LNE) and lean-elite (LE) group completed the 10-week training program. Drop out reasons were muscular injuries and respiratory tract infections. Average training mileage was 35 ± 8 km/week in ONE, 38 ± 1 km/week in LNE, and 54 ± 2 km/week in LE during the 10 weeks of the training program. The difference in distance between ONE/LNE and LE was significant (*P* < .01). 

Participants in all three groups showed a reduction in total body weight (ONE −1.4 kg, LNE −1.1 kg and LE −1.7 kg; *P* < .05) and corresponding BMI (Table 1). In ONE and LE, weight loss reflected a decrease of the waist circumference (ONE −2.8 cm; LE −1.9 cm; *P* < .05). A significant reduction in total body fat was only found in LNE (*P* < .01) and LE (*P* < .05). Body fat reduction in obese subjects failed to reach significance (*P* = .058) (Table 1).

At baseline, individual anaerobic threshold (IAT) was lowest in ONE (10.6 ± 0.8) compared to LNE (11.6 ± 0.9) and LE (13.6 ± 1.4). Exercise training resulted in a significant improvement of physical fitness (IAT) in all groups, which was most pronounced in ONE (*P* < .01) (Table 1).

We also analyzed the changes of leukocytes, monocytes, and platelets between before and after the training. There were no significant changes in leucocytes or monocytes, we only found a significant change for the thrombocytes in response to exercise. Thrombocytes increased by 8000/mm^3^, which we think is not a physiologic relevant finding (data not shown).

### 3.2. Flow-Cytometer Analysis of Peripheral Blood Mononuclear Cells

BDCA-1/-2 positive cells represent approximately 1% of the circulating leucocytes. Circulating BDCA-1+ DCs are reduced in cerebral- and myocardial-infarction and are associated with increased occurrence of infections [17, 18].

**Figure 1:**
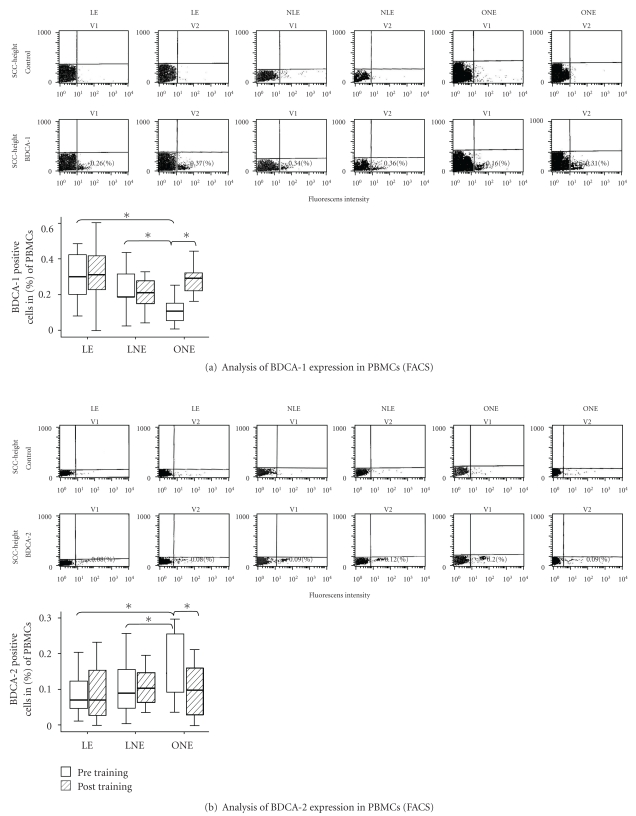
Representative FACS scans in PBMC expressing of BDCA-1 (a) and BDCA-2 (b) in LE, LNE, and ONE at baseline and after training. Data presented state the expression of the corresponding receptors in percent. Statistical analysis of the different timepoints (intra group analysis) was performed using the Wilcoxon Test; the comparison of the different groups with each other (inter group analysis) was done by using the Mann-Whitney *U* Test (*P < .05; *n* = 15/16/16).

 Myeloid DCs (BDCA-1+) at baseline were reduced in ONE compared to lean subjects. At baseline, BDCA-1 expression was 0.15% in the ONE (*P* < .05 versus LNE/NE) and increased to 0.30% (+100%; *P* < .05) after the training period. Exercise training had no significant effects on BDCA-1 expression in LNE and LE (LNE 0.27%/0.26%; LE 0.33%/0.35%) (Figure 1(a)).

The median difference of BDCA1-levels before and after training was significantly different between the three groups (*P* = .039). This effect was mainly seen between LNE and ONE (−0.05% versus +0.17%, *P* = .006, supplementary Table I available on line at doi: 10.1155/2011/308965).

Plasmacytoid DCs (BDCA-2+) at baseline were significantly higher in ONE compared to LE and LNE. There was no significant difference between LNE (0.11%) and LE (0.09%). After the training program, BDCA-2 expression in ONE decreased significantly (−61 %; *P* < .05) and reached the levels of LNE (0.12%) and LE (0.09%), which remained unchanged after the exercise intervention (Figure 1(b)).

There were no significantly different effects among the three groups (*P* = .117, supplementary Table II available on line at doi: 10.1155/2011/308965).

### 3.3. RT-PCR for Toll-Like-Receptors (TLRs)

TLRs recognize so-called pathogen associated molecular patterns (PAMPs) and thereby activate immune pathways. TLRs have an essential role in the innate immune recognition of microorganisms.

TLR2 recognizes components from a variety of microbial pathogens. These include lipoproteins from pathogens such as Gram-negative bacteria [19, 20].

**Figure 2:**
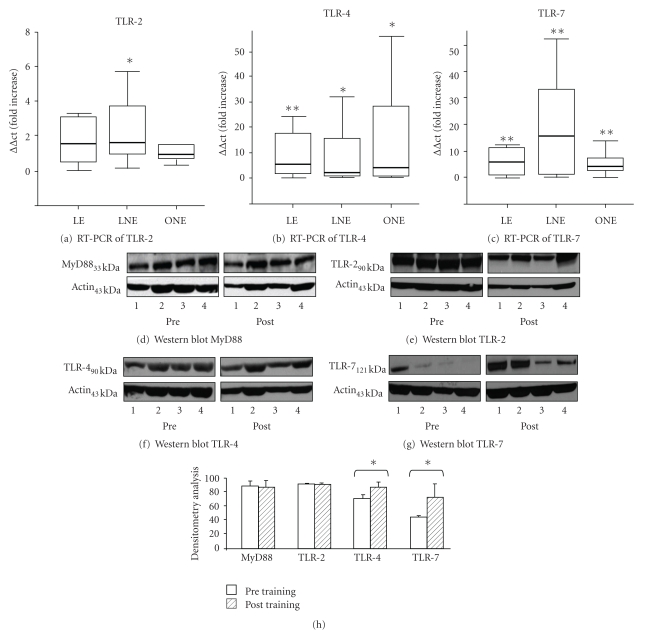
Expression of TLR-2 (a), TLR-4 (b), and TLR-7 (c) by analysis of mRNA levels via RT-PCR. Training-induced changes were analyzed using the delta-delta ct method. Expression of MyD88 (d), TLR-2 (e), TLR-4 (f), and TLR-7 (g) by analyses of protein expression (Western blot of 4 representative examples of the LNE-group). Densitometry analysis (h) found a significant increased expression for TLR-4 and TLR-7. For MyD88 and TLR-2, no significant changes were found. Statistical analysis of the different time-points (intra group-analysis) was performed using the Wilcoxon-Test; the comparison of the different groups with each other (inter group analysis) was done by using the Mann-Whitney *U* Test (**P < .01; *P < .05; *n* = 15/16/16 for PCR and *n* = 8 for Western blot).

TLR-2: At baseline, no difference in TLR-2 was found within the groups (data not shown). Gene-expression increased significantly in response to exercise in LNE (1.7-fold increase; *P* < .05). TLR-2 showed similar patterns in the other groups without reaching significance (LE 2.3-fold increase; ONE 0.9-fold increase) (Figure 2(a)).

TLR4 is essential for the signalling of LPS, with is a major component of the outer membrane of Gram-negative bacteria [19, 20].

TLR-4: TLR-4 at baseline did not differ within the groups (data not shown). TLR-4 was significantly upregulated in all three groups in response to exercise. The differences were most pronounced in LE (increase more than 10-fold; *P* < .01). TLR-4 increase was 9.6 fold in LNE (*P* < .05) and 14 fold in ONE (*P* < .05) (Figure 2(b)).

TLR7 has been shown to recognize the single-strand RNA and double-strand RNA of virus-origin such like influenza viruses or Respiratory syncytial viruses [19, 20].

TLR-7: TLR-7 at baseline did not differ within the groups (data not shown). All three groups showed a highly significant exercise-induced upregulation of TLR-7 (LE: 14.9-fold increase; LNE: 22-fold increase; ONE: 6.3-fold increase; all *P* < .01) (Figure 2(c)). 

MyD88 is a universal adapter protein as it is used by TLR-2,-4, and -7 to activate the transcription factor  NF-*κ*B [20].

For TLR-2,-4, and -7, there were no significantly different effects among the three groups (*P* = .450, *P* = .712, and *P* = .235, supplementary Table III available on line at doi: 10.1155/2011/308965).

MyD88: MyD88 at baseline did not differ within the groups (data not shown). All three groups showed no significant exercise-induced changes in MyD88 gene-expression. (LE: 2.7 ± 1.47-fold increase; LNE: 2.21 ±  0.92-fold increase; ONE: 2.11 ± 0.79 fold increase; n.s.) (data not shown). 

NF*κ*B: NF*κ*B at baseline did not differ within the groups (data not shown). All three groups showed no significant exercise-induced changes in *NF*κ*B *gene expression. (LE: 1.28 ± 0.36-fold increase; LNE: 1.71 ± 0.41-fold increase; ONE: 1.43 ± 0.5-fold increase; all *P* < .01) (data not shown) [23].

### 3.4. Western Blot

To demonstrate a relationship between gene activation and protein expression, we performed Western blot analysis for TLR-2/-4/-7 and MyD88 representative for 8 probands of the LNE (Figures 2(d)–2(h)) group. For TLR-4 and TLR-7, we found a significant increase (+22.6/+63.3%) in protein expression (both *P* < .05). No changes in protein expression were found for TLR-2, and MyD88.

### 3.5. ELISA for oxLDL, Adiponectin, IL-6-, and TNF-*α*

***oxLDL*** is a principal form of cholesterol that accumulates in atherosclerotic lesions or plaques. Its levels are generally considered to be associated with an increased atherosclerotic burden. At baseline, circulating oxLDL levels were significantly higher in ONE (90 ng/mL; *P* < .05) compared to LE (77 ng/mL). After exercise training, oxLDL levels decreased by –27% (66 ng/mL; *P* < .05) in ONE. In contrast to this finding, we found an increase in oxLDL after training in LE (273 ng/mL; *P* < .05) and LNE (186 ng/mL), the latter did not reach significance (Table 1). The median difference before and after training was significantly different between the three groups (*P* = .001). This effect was mainly seen between LNE and ONE (+41 ng/mL versus −35 ng/mL, *P* = .009) and between LE and ONE (+98 ng/mL versus −35 ng/mL, *P* = .001, supplementary Table III available on line at doi: 10.1155/2011/308965).

At baseline, adiponectin levels were highest in LE (7642 ng/mL), followed by LNE (5972 ng/ml) and lowest in ONE (5459 ng/mL). The difference between LE and ONE was significant (*P* < .05). Exercise training induced an increase of adiponectin serum levels in all groups (LE 8220 ng/mL; LNE 7713 ng/mL; ONE 5964 ng/mL), reaching significance in LNE only (*P* < .05) (Table 1). There were no significantly different effects among the three groups (*P* = .335, supplementary Table III available on line at doi: 10.1155/2011/308965).

***IL-6*** is secreted by T-cells and macrophages. It stimulates the immune response and is released by skeletal muscles in response to endurance exercise. The ELISA-based analysis of IL-6 at baseline detected the lowest levels in LE (0.72 pg/mL). In comparison, IL-6 concentrations of LNE and ONE were significantly higher (1.43 pg/mL and 1.24 pg/mL resp.; *P* < .01). Training induced a slight upregulation of IL-6 in LNE (1.52 pg/mL) and ONE (1.68 pg/mL), whereas IL-6 levels further decreased in LE (0.44 pg/mL). These changes were not significant (Table 1). The median difference before and after training was significantly different between the three groups (*P* = .048). This effect was mainly seen between LE and LNE (−0.12 pg/ml versus +11 pg/ml, *P* = .013, supplementary Table III available on line at doi: 10.1155/2011/308965).

**TNF-*α*** is a cytokine and mediator of systemic inflammation. It is involved in acute phase reactions. LE showed the lowest TNF-**α** levels (0.55 pg/mL) at baseline, with the highest levels being detected in ONE (1.62 pg/mL) and in LNE (1.19 pg/mL). Exercise training generated a downregulation of TNF-**α**serum levels in all groups (LE: down to 0.22 pg/mL; ONE: down to 1.12 pg/mL and LNE: down to 0.77 pg/mL), without reaching statistical significance (Table 1). There were no significantly different effects among the three groups (*P* = .821, supplementary Table III available on line at doi: 10.1155/2011/308965).

## 4. Discussion

Exercise and obesity have been shown to modify the immune system [1, 12]. In this paper we focused on the effect of regular aerobic exercise on markers of the innate immune-response [26]. All subjects, including the obese athletes, were pretrained, and so these obese individuals are not representative for the majority of obese subjects. 

We were able to demonstrate a difference in the expression of the BDCA-1 and -2 fractions in lean and obese subjects and, for the first time, illustrate the effect of exercise on dendritic cell subpopulations and TLR-2/-4, and -7 expressions.

In obese subjects, exercise induced an upregulation of BDCA-1+ cells, whereas BDCA-2+ cells were downregulated. We further found an increased gene and protein expression of TLR-4 and TLR-7 independent of the body composition of the athletes. With respect to the signaling pathway, we did not find proof of an activation of the TLR pathway, since MyD88 gene activation and protein expression remained unchanged during training. This is supported by the fact that the NF-kB transcription faction mRNA was not upregulated over time. 

There is evidence that lower levels of circulating BDCA-1 DCs in obese subjects are associated with obesity-induced dyslipidemia [27]. Dyslipidemia is not restricted to the routinely quantified lipids such as LDL, HDL, and triglyceride but enfolds biomarkers such as adiponectin and oxLDL [28]. 

Robertson et al. demonstrate a distinct [29] Th-2-based autoimmune response to the autoantigen malondialdehyde-LDL [30], providing evidence for dyslipidemia influencing the immune response. Furthermore, previous studies have shown that dyslipidemia may impair antiviral, and antibacterial responses [31–34] which may, in part, explain the increased cardiovascular risk and mortality in obese individuals [8]. However, the mechanisms have yet to be fully identified. It remains unclear whether dyslipidemia leads to an overall immunosuppression or whether it modulates specific immune pathways. 

In previous studies, Th-1-induced activation of DCs was considerably impaired upon oxLDL treatment, whereas Th-2-induced development was enhanced [31–34]. In our study, obese subjects showed high oxLDL levels accompanied by the lowest BDCA-1 expressing cells. During training, oxLDL decreased whereas BDCA-1 positive cells increased. Fuchs et al. described a reduction of BDCA-1 levels in patients with cerebral infarction, which was associated with higher infection rates. When analyzing previous data with respect to BDCA-1 expression, an increase of BDCA-1 expression seems to coincide with a higher antiviral, antibacterial and antitumor activity [35–37].

Other animal studies support the beneficial effect of exercise on dendritic cells by demonstrating an enhanced activity of DCs against tumor and pathogen-eliminating cells [38–40]. There is also evidence that demonstrates an association between decreased DCs and the progression of coronary artery disease [41]. The exercise-induced reduction of oxLDL levels in obese participants, as presented in our study, might diminish the susceptibility of low-density lipoprotein to oxidation which in turn may prevent endothelial dysfunction and reduce inflammation [42, 43]. This does not, however, explain the increase in oxLDL seen in LE, which did not correlate with changes in BDCA-1/-2 expression. Further aspects seem to influence this immunological relation. LE are characterized by very high training intensities and duration which might cause high levels of oxidative stress leading to an upregulation of oxLDL. 

In addition to the changes in immune cell subpopulations, we were also able to detect effects related to the expression of TLRs. TLR-2 is responsible for the detection of gram-positive bacteria, TLR-4 for gram-negative bacteria, and TLR-7 has a high detection potential for ssRNA (e.g., influenza virus) [19]. Besides anti-inflammatory effects, TLR-4 and -7 upregulation induce antitumor activity [44]. 

The exercise program induced an upregulation of the gene and protein expression of TLR-4 and TLR-7. These could be interpreted as an increased immune responsiveness and would point out the strenuous nature of our training regimen. 

To date, there are only a handful of studies that analyze the effect of exercise on TLRs. Stewart and Flynn et al. described an exercise-induced decrease of TLR-4 on CD14 positive cells [45, 46]. The discrepancy to our findings can be explained by the fact that we analyzed total PBMCs, whereas the above working group looked at CD14+ cells only. Looking at CD14 cells primarily does not take into account that TLRs are also expressed on B-,T-cells and naturally on DCs [47]. 

The upregulation of TLRs may be accompanied by an increased expression of sentinel receptors. This may implicate a potentially higher capacity in the first line response to a potential pathogen. The TLR modifications cannot be validated as an increased inflammatory response, since systemic cytokines were stable or even decreased after the training program. Furthermore, the lack of MyD88 and NF-kB upregulation implicates that the TLR activation is not induced by an inflammatory stimulus.

In our study setting, exercise did not significantly alter classic mediators of inflammation. This is probably due to the fact that the subjects in our study were already physically trained before the start of the training program and showed relatively low inflammatory markers at baseline, a condition that has been previously described [48]. Main differences were seen in ONE compared to LE. A main discrepancy between these two groups is the waist circumference, which correlates well with the total body fat burden. There is also a correlation between fat-tissue-mass and hormone activity. oxLDL and adiponectin levels differed in ONE and LE at baseline. Exercise training reduced waist circumference and oxLDL levels in ONE, which was associated with increased adiponectin levels. Statistical differences between ONE and LNE could not be detected.

## 5. Conclusions

Exercise-induced immunomodulation such as changes in BDCA-1 and -2 and TLR expression in obese subjects seems to be consistent with a reduction of total body fat and dyslipidemia. We describe new immunomodulatory findings by which regular aerobic exercise modulates the immune system response, increases inflammatory resistance, and mediates cardiovascular protection in obesity. Future studies are necessary to determine to what extend these immunomodulatory effects influence the cardiovascular system.

## 6. Limitations

As in most studies involved in exercise intervention, study group size is limited. 13 subjects dropped out during the exercise period. All participants prepared to run a marathon at the end of the 10-week training program. All subjects, including the obese athletes, were pretrained, and so we did not include the sedentary obese population. However, from what is currently known in exercise immunology, one may speculate that the effect of the training intervention would likely be more pronounced in sedentary obese subjects. 

 Professional exercise supervision was offered throughout the trial. Training intensity was high in all groups and was adapted depending on individual training levels. Regular day-to-day exercise with lower training intensity is likely to show similar results in previously untrained obese subjects. Larger studies including physically inactive obese individuals undergoing less sophisticated training protocols are necessary to determine achievable results. Moreover, to achieve more mechanistical insight future studies should incorporate immunomodulatory effects of exercise on microvascular function in particular.

## Supplementary Material

Supplementary Table I: Kruskal-Wallis analysis of the medians for BDCA1/-2.
Supplementary Table II: Kruskal-Wallis analysis of the medians for TLR-2,-4,-7.
Supplementary Table III: Kruskal-Wallis analysis of the medians for oxLDL, adiponectin, IL-6 and TNF-*α*.

## Figures and Tables

**Table 1:** Clinical characteristics such as age, weight, BMI, waist circumference (WC), body fat, and individual anaerobic threshold (IAT) in all probands including baseline and posttraining parameters. Measured cytokines-serum levels in pg/ml or ng/ml include pre training and post training parameters. Cytokines in all groups decreased compared to pre training. Significant *P* values are marked in bold. Statistical analysis of the different time-points (intra group-analysis) was performed using the Wilcoxon Test; the comparison of the different groups with each other (inter group analysis) was done by using the Mann-Whitney *U* Test.

	ONE (*n* = 15) age 40 ± 6 years <40 km/week			LNE (*n* = 16) age 40 ± 6 years <40 km/week			LE (*n* = 16) age 40 ± 7 years >55 km/week		
Clinical characteristics	Pre	Post	*P*	Pre	Post	*P*	Pre	Post	*P*
Weight (kg)	99 ± 11.9	97.6 ± 12.2	**<**.05	79.6 ± 8	78.5 ± 9	**<**.05	75.7 ± 11.5	74.4 ± 11	**<**.05
BMI (kg/m^2^)	30 ± 2	29 ± 2	**<**.05	25 ± 2	24 ± 2	.06	23 ± 2	22 ± 1	**<**.01
WC (cm)	106 ± 5	103 ± 7	**<**.05	87 ± 8	86 ± 7	.47	83 ± 8	81 ± 7	**<**.05
Body Fat (%)	27 ± 3	24 ± 3	.058	17 ± 5	15 ± 4	**<**.01	13 ± 4	11 ± 2	**<**.05
IAT (km/h)	10.6 ± 0.8	11.1 ± 1	**<**.01	11.6 ± 1	12,1 ± 1	**<**.01	13.6 ± 1.4	14.1 ± 1	**<**.01

Cytokines-serum levels									

TNF-*α* (pg/mL)	1.62 ± 3.1	1.12 ± 2.3	n.s.	1.19 ± 2.9	0.77 ± 2.1	n.s.	0.55 ± 1.7	0.22 ± 0.8	n.s.
IL-6 (pg/mL)	1.43 ± 1.5	1.68 ± 1.4	n.s.	1.24 ± 1.8	1.52 ± 2.4	n.s.	0.72 ± 0.5	0.44 ± 0.3	**<**.05
oxLDL (ng/mL)	272.6 + 403.0	65.5 + 57.3	**<**.05	83.1 + 39.3	186.3 + 202.7	n.s.	76.6 + 41.4	243.9 + 204.4	**<**.01
Adiponectin (ng/mL)	5465.5 + 2999	5932.5 + 3131	n.s.	5972.2 + 2999	7355.1 + 3399	n.s.	7642.4 + 3574	8219.6 + 3855	n.s.

## Abbreviations

ACSM: American College of Sports Medicine
BDCA-1, -2: Blood dendritic cell antigen -1, -2
Bis-TRIS-gel: Bis(2-hydroxyethyl)amino-tris(hydroxymethyl)methan-gel
BMI: Body Mass Index
CD14: Cluster of differentiation
cDNA: Complementary Deoxyribonucleic acid
CRP: C-reactive protein
DC: Dendritic cells
ECL-Pierce: Enhanced Chemiluminescent-Pierce
ELISA: Enzyme-linked immunosorbent assay
FACS: Fluorescence-activated cell sorting
FCS: Fetal calf serum
FITC: Fluoreszeinisothiocyanat
GAPDH: Glyceraldehyde 3-phosphate dehydrogenase
HDL: High-density lipoprotein
HRP: Horseradish peroxidase
IAT: Individual anaerobic threshold
IL-6, -8, -10: Interleukin
LDL: Low-density lipoprotein
LE: Lean elite
LNE: Lean nonelite
MIP: Macrophage inflammatory protein
mRNA: Messenger Ribonucleic acid
MyD88: Myeloid differentiation primary response gene 88
NF-*κ*B: Nuclear factor kappa-light-chain-enhancer of activated B cells
ONE: Obese non elite
oxLDL: Oxidized low-density lipoprotein
PAMPs: Pathogen-associated molecular patterns
PBMCs: Peripheral blood mononuclear cells
PBS: Phosphate buffered saline
PCR: Polymerase Chain Reaction
PE: Phycoerythrin
PI: Propidium iodide
RIPA-buffer: Radioimmunoprecipitation assay buffer
RT-PCR: Reverse transcriptase Polymerase Chain Reaction
SDM: Standard-deviation of the mean
SDS-PAGE: Sodium dodecylsulfate polyacrylamide gel electrophoresis
ssRNA: Single-stranded Ribonucleic acid
TLR-2, -4, -7: Toll-like-receptors
TNF-*α*: Tumor necrosis factor alpha
WC: Waist circumference.
