# Supplementary material for: Immunomodulatory Effects of Aerobic Training in Obesity
Source: Mediators Inflamm. 2011 Mar 10;2011:308965. doi: 10.1155/2011/308965 (PMC3065046; doi:10.1155/2011/308965)
Supplement: Supplementary file 1 — Supplementary Table I: Kruskal-Wallis analysis of the medians for BDCA1/-2. Supplementary Table II: Kruskal-Wallis analysis of the medians for TLR-2,-4,-7. Supplementary Table III: Kruskal-Wallis analysis of the medians for oxLDL, adiponectin, IL-6 and TNF-α. [file 308965.f1.pdf]

supplemental Table I

	BDCA1			BDCA2		
	LE	LNE	ONE	LE	LNE	ONE
**median (before training)**	0.30	0.25	0.11	0.07	0.09	0.15
**median (after training)**	0.31	0.23	0.29	0.07	0.10	0.10
**p (Wilcoxon-test: median before/ after training)**	0.796	0.379	**0.019**	0.865	1.000	**0.038**
**median difference**	0.08	-0.05	0.17	0.00	-0.01	-0.04
**p (Kruskal-Wallis-H-test of median difference: LE, LNE , ONE)**	**0.039**			0.117		
	**p (Mann- Whitney-U-test)**	**LE-LNE: 0.374<br>LNE-ONE: 0.006<br>LE-ONE:0.188**				
