# Supplementary material for: Immunomodulatory Effects of Aerobic Training in Obesity
Source: Mediators Inflamm. 2011 Mar 10;2011:308965. doi: 10.1155/2011/308965 (PMC3065046; doi:10.1155/2011/308965)
Supplement: Supplementary file 2 [file 308965.f2.pdf]

supplemental Table II

	TLR 2			TLR 4			TLR 7		
	**LE**	**LNE**	**ONE**	**LE**	**LNE**	**ONE**	**LE**	**LNE**	**ONE**
**median (??ct)**	1.33	1.57	0.91	5.28	1.74	4.00	5.66	16.00	4.78
**minimum**	0.01	0.13	0.31	0.01	0.23	0.15	0.03	0.33	0.22
**maximum**	8.00	9.19	8.57	42.22	45.25	55.72	64.00	84.45	24.25
**p (Wilcoxon -test)**	0.158	**0.026**	0.432	**0.003**	**0.027**	**0.016**	**0.009**	**0.002**	**0.002**
**p (Kruskal-Wallis-H-test)**	0.450			0.712			0.235		
