# Supplementary material for: Immunomodulatory Effects of Aerobic Training in Obesity
Source: Mediators Inflamm. 2011 Mar 10;2011:308965. doi: 10.1155/2011/308965 (PMC3065046; doi:10.1155/2011/308965)
Supplement: Supplementary file 3 [file 308965.f3.pdf]

supplemental Table III

	TNF–a (pg/ml)			Il – 6 (pg/ml)			ox-LDL (ng/ml)			Adiponectin (ng/ml)		
	LE	LNE	ONE	LE	LNE	ONE	LE	LNE	ONE	LE	LNE	ONE
**median (before training)**	0.00	0.00	0.00	0.54	0.64	0.83	60.68	80.49	104.40	6512.5	5463.0	4957.5
**minimum (before training)**	0.00	0.00	0.00	0.08	0.11	0.00	39.33	32.12	48.69	3408	2117	1364
**maximum (before training)**	6.80	9.72	11.07	1.95	7.95	5.40	175.68	143.56	1313.2	15916	10392	11795
**median (after training)**	0.00	0.00	0.00	0.44	0.95	1.19	210.94	161.28	47.23	7325.5	7092.5	5648.0
**minimum (after training)**	0.00	0.00	0.00	0.00	0.14	0.01	22.83	29.63	0.13	3386	2734	833
**maximum (after training)**	3.47	7.26	8.85	1.65	10.5	5.22	732.92	773.67	192.35	15849	12986	10863
**p (Wilcoxon-test : median before/ after**	0.59	0.50	0.86	**0.023**	0.17	0.47	**0.006**	0.086	**0.041**	0.301	**0.02**	0.47
**median difference**	0.00	0.00	0.00	-0.12	0.11	0.21	98.36	40.54	-34.51	265.00	1062.0	-465.5
**p (Kruskal-Wallis-H-test of median difference: LE, LNE , ONE)**	0.821			**0.048**	**p (Mann-Whitney-U-test)**	**LE-LNE: 0.013<br>LNE-ONE: 0.901<br>LE-ONE: 0.096**	**0.001**	**p (Mann-Whitney-U-test)**	**LE-LNE: 0.256<br>LNE- ONE: 0.009<br>LE-ONE: 0.001**	**0.335**		
